# A significant role for tumor necrosis factor in *Nlrp3* inflammasomeopathies

**DOI:** 10.1186/1546-0096-13-S1-O27

**Published:** 2015-09-28

**Authors:** M McGeough, A Wree, C Pena, M Inzaugarat, A Feldstein, H Hoffman

**Affiliations:** 1 University of California San Diego, Pediatrics, La Jolla, USA; 2 RWTH University Hospital Aachen, Internal Medicine III, Aachen, Germany

## Introduction

The NLRP3 inflammasome is a protein complex responsible for caspase-1 dependent maturation of the pro-inflammatory cytokines IL-1β and IL-18. Gain of function missense mutations in NLRP3 cause the disease spectrum known as cryopyrin-associated periodic syndromes (CAPS).

## Objective

To elucidate systemic autoinflammatory disease mechanisms involved NLRP3 inflammasomeopathies other than IL-1β and IL-18.

## Materials and methods

Knock-in *Nlrp3^L351P/+^/CreL/Il1b-/-/Il18-/-*mice (*FCAS Il1b^-/-^Il18-/-*), *Nlrp3^L351P/+^/CreL/* Casp1^-/-^, (*FCAS Casp1*^-/-^),*Nlrp3^A350V/+^/CreL (MWS)* and *Nlrp3^A350V/+^/CreL/Tnf^-/-^ (MWS Tnf^-/-^)* were generated in which Cre-mediated expression is limited to the myeloid cell lineage.

## Results

Nearly all *FCAS Il1b^-/-^Il18-/-*mice survived and grew normally until adulthood however, investigation of mice at > 6 months of age showed marked splenomegaly and elevated numbers of white blood cells as compared to *FCAS Casp1^-/-^*mice and non-mutant *Il1b^-/-^Il18-/-*littermates, suggesting a caspase-1 dependent phenotype independent of IL-1β and IL-18. To examine other potential inflammatory mediators, non-lethal *in vivo* LPS (5 ug/g) stimulation of FCAS Il1b^-/-^Il18^-/-^mice revealed significantly elevated levels of serum TNF at both 2 and 6 hours post induction when compared to *FCAS Casp1^-/-^*mice and non-mutant *Il1b^-/-^Il18-/-*controls. To further investigate the role of TNF in *Nlrp3* inflammasomeopathies, *MWS* mice (which die within two weeks of birth) were bred on a TNF knockout background. MWS Tnf^-/-^ pups were indistinguishable from non-mutant controls with all animals surviving to adulthood with normalization of both body and spleen/body weight comparisons. Serum analysis of *MWS Tnf^-/-^* pups showed attenuation of NLRP3 inflammsome related cytokines when compared to intact *MWS* pups. The skin of intact *MWS* pups exhibited strong neutrophilic and inflammatory macrophage infiltrations, which were normalized in *MWS Tnf*^-/-^ animals. Interestingly, *MWS Tnf ^+/-^* pups also showed an intermediate protective effect on all of the aforementioned comparisons. To determine if TNF ablation could be recapitulated with therapeutic intervention, *MWS* pups were treated with recombinant soluble TNF receptor (Etanercept 400ug/g sc EOD). Treatment provided similar phenotypic rescue and extended survival for an average of 22 days after cessation of treatment. Adult *MWS Tnf ^-/-^*mice at > 6 months of age were found to have splenomegaly and elevated numbers of white blood cells when compared to non-mutant *Tnf ^-/-^*littermates, implicating a role for other inflammatory mechanisms as mice age.

## Conclusion

TNF plays an unexpected and significant role in murine inflammasomeopathies, which may have therapeutic implications for CAPS patients with incomplete responses to IL-1 targeted therapies.

